# Impact of social participation on health among middle-aged and elderly adults: evidence from longitudinal survey data in China

**DOI:** 10.1186/s12889-020-08650-4

**Published:** 2020-04-15

**Authors:** Xinxin Ma, Xiangdan Piao, Takashi Oshio

**Affiliations:** 1 Faculty of Social Sciences, University of Toyama, Gofuku 3190, Toyama, 930-8555 Japan; 2 School of Engineering, Kyushu University, 744 Motooka, Nishi-ku, Fukuoka, 819-0395 Japan; 3 Institute of Economic Research, Hitotsubashi University, 2-1, Naka, Kunitachi, Tokyo, 186-8603 Japan

**Keywords:** China, Health outcomes, Middle-aged and elderly, Lagged variables, Social participation

## Abstract

**Background:**

Social participation (SP) is known to have a favourable impact on health. However, studies on this issue have been conducted mainly in advanced countries, and results in China have been mixed. This study examined the impact of SP on health outcomes of middle-aged and elderly adults in China, adjusted for simultaneity and heterogeneity biases.

**Methods:**

In total, 57,417 observations of 28,935 individuals obtained from the population-based, three-wave panel survey, Chinese Health and Retirement Longitudinal Study (CHARLS), conducted in 2011, 2013, and 2015 were used. The associations between one- or two-wave-lagged SP and health outcomes (mental health, self-rated health [SRH], activities of daily living [ADL], and diagnosed diseases) were examined by linear regression models. Individual-level heterogeneity was addressed by the random-effects estimation method.

**Results:**

SP was found to have a positive impact on mental health and ADL. Specifically, one-wave-lagged SP improved mental health measure (range: 10–70) by 0.820 (standard error [SE]: 0.199, *p* <  0.001), the basic ADL measure (range: 6–24) by 0.147 (SE: 0.043, *p* <  0.001), and the instrumental ADL measure (range: 5–20) by 0.159 (SE: 0.035, *p* <  0.001). In contrast, SP did not significantly affect SRH or diagnosed diseases. The impact of SP differed by SP type; playing Mah-jong (Chinese traditional game), chess, or cards, or going to the community club had the most favourable effect. The impact of SP on health was also greater for women than men and greater for individuals aged 60–69 years than those aged 45–59 years and aged 70 and older.

**Conclusions:**

SP had a positive, albeit selective, impact on health outcomes among middle-aged and elderly adults in China. The results suggest that policy measures to encourage these individuals to engage in SP are needed to enhance their health.

## Background

Social participation (SP) is known to have a preventive impact on illness, particularly for the elderly. Several studies have demonstrated that SP experiences prevent the onset of diseases and functional disability [1–5], mental health disorder [6–11], cognitive impairment [3, 7, 12], and delayed mortality [13, 14] among the elderly. Interactions with others in society or a community—considered core aspects of SP and closely related to the concept of hedonic stock—are expected to accelerate adaptation to health shocks, as well as prevent their onsets [15].

Compared to studies on the issue in developed countries, China’s studies have been relatively scarce and provide mixed results. Studies have found that social network and social capital—which are concepts closely related to and overlapped with SP—were positively associated with health outcomes [16–19], whereas one study found no association between social capital and geriatric depression [20]. Regarding SP, studies have shown conflicting evidences; SP was found to improve self-rated health (SRH) and mental health but have no effect on chronic diseases [21], whereas an inverse association between SP and the onset of hypertension was found for women but not for men [22]. Meanwhile, one study showed that SP was associated with a reduced risk for the onset of functional disability among elderly Chinese [23].

Following these preceding studies, this study investigated the impacts of SP on health outcomes among middle-aged and elderly adults, using the longitudinal survey data in Chinese. The main contributions of this study are as follows. First, the reverse causality and heterogeneity problems, which may have caused mixed results in preceding studies, were addressed by the lagged variable (LV) method and random effects (RE) models. Second, the results were compared across various health outcomes, rather than focusing on any specific type, to evaluate the overall impact of SP on health. Third, the results were compared across different demographic groups, considering their possible heterogeneity. These results are expected to provide new insights into the associations between SP and health and help us compare the observations in China with those in other countries.

## Methods

### Study sample

The three-wave longitudinal data obtained from the Chinese Health and Retirement Longitudinal Study (CHARLS) – which was conducted by Peking University representative regions in China in 2011, 2013, and 2015 – were used in this study. The survey objects were individuals aged 45 and older. The baseline wave included about 10,000 households and 17,500 individuals in 150 counties/districts and 450 villages/resident committees. CHARLS contained a rich set of individual-level information, such as demographic characteristics, family structure, household consumption, social participation situation, subjective and objective health status, and other related information.

In this study, the individuals who were aged 45 and older in the baseline survey, and remained in at least one of two follow-up surveys, were focused on. Furthermore, for the analysis about the onset of each diagnosed disease, individuals with an established diagnosis were removed. After further excluding the respondents who were missing key variables used in statistical analysis, the number of individuals used in this study ranged from 5986 to 7009, depending on health outcomes and estimation models.

The CHARLS dataset, which was used in this study, was publicly available, and the Ethical Review Committee of Peking University in China approved its study protocol. Hence, the ethical approval was not needed for this study.

### Measures

The key independent variable was SP. As the dependent variables, four types of health outcomes were considered: (1) mental health, (2) SRH, (3) diseases, and (4) activities of daily living (ADL).

#### Social participation (SP)

Regarding SP, CHARLS asked respondents, ‘Have you done any of these activities in the last month?’, listing seven types of social activities: (a) interacting with friends, (b) playing Mah-jong, chess, cards, or going to the community club, (c) providing help to family, friends, or neighbours who do not live with you and did not pay for your help, (d) going to a sport, social, or other club activity; (e) participating in a community-related organization, (f) doing volunteer or charity work, and (g) caring for a sick or disabled adult who does not live with you and did not pay for your help. Seven binary variables of each SP activity were constructed by allocating ‘1’ to the answer *yes* and ‘0’ otherwise. A binary variable of overall SP was constructed by allocating ‘1’ to those participating in at least one type of SP activity and ‘0’ to others.

#### Mental health

Two types of mental health scores, MH1 and MH2, were constructed as follows. First, based on the questionnaire, ‘How would you rate your memory at the present time? Would you say it is excellent, very good, good, fair, or poor?’, MH1 was scored as follows: *excellent* = 5, *very good* = 4, *good* = 3, fair = 2, and *poor* = 1. Another mental health score, MH2, was constructed based on answers to ten questions of the Center for Epidemiologic Studies Depression Scale (CES-D), the validity of which has been confirmed among elderly Chinese [24]. Specifically, CHARLS provided ten items referring to feeling and behaviour about mental health status during the previous week: (a) ‘I was bothered by things that don’t usually bother me’, (b) ‘I had trouble keeping my mind on what I was doing’, (c) ‘I felt depressed’, (d) ‘I felt everything I did was an effort’, (e) ‘I felt hopeful about the future’, (f) ‘I felt fearful’, (g) ‘My sleep was restless’, (h) ‘I felt lonely’, (i) ‘I could not get “going”’, and (j) ‘I was happy’. For each item, respondents’ responses were scored as *rarely or none of the time (< 1 day)* = 7, *some or a little of the time (1–2 days)* = 5, *occasionally or a moderate amount of the time (3–4 days)* = 3, *most or all of the time (5–7 days)* = 1, while the score was reversed for (e) and (j). The scores for ten items were summed up and defined as MH2 (range: 10–70). The Chronbach’s *α* was 0.809 in the current study sample, indicating a reasonable level of internal consistency. For both MH1 and MH2, a higher value means better mental health.

#### Self-rated health (SRH)

Based on the respondents’ responses to the question about SRH, a five-point score variable of SHS was constructed as *very good* = 5*, good* = 4, *fair* = 3, *poor* = 2 and *very poor* = 1. A higher value means better SRH. Alternatively, a binary variable of SES was constructed as 1 = *very good* and *good*, 0 = *poor* and *very poor*, but logistic regression models with it obtained similar results. Hence, only the results with a five-point score value of SRH were reported in what follows.

#### Activities of daily living (ADL)

CHARLS provided information about two types of ADL: the basic activities of daily living (BADL) and the instrumental activities of daily living (IADL). CHARLS asked the respondents whether they have any difficulty in doing each of the following activities: (a) dressing, (b) bathing, (c) eating, (d) getting into or out of bed, (e) using the toilet, and (f) controlling urination and defecation for BADL. They were also asked about the level of difficulty when (a) doing household chores, (b) preparing hot meals, (c) shopping for groceries, (d) taking the right portion of medication on time, and (e) managing money for IADL. For both BADL and IADL, the respondents’ responses were scored as: *I don’t have any difficulty* = 4, *I have difficulty but can still do it* = 3, *I have difficulty and need help* = 2, *I cannot do it* = 1. The scores were summed up and defined as BADL (range: 6–24) and IADL (5–20). The Chronbach’s *α* was 0. 833 for BADL and 0.816 for IADL in the current study sample, both indicating reasonable levels of internal consistency. For both variables, a higher value means fewer difficulties in daily living.

#### Diseases

Based on the questionnaire item, ‘Have you been diagnosed with diseases by a doctor?’, seven binary variables of diagnosed diseases were constructed: (a) hypertension or dyslipidaemia, (b) diabetes or high blood sugar, (c) heart attack or stroke, (d) cancer or malignant tumour, (e) emotional, nervous, psychiatric problems or memory-related disease, (f) stomach or other digestive disease, and (g) other disease.

#### Covariates

A set of various covariates regarding (1) demographic and family structure, (2) socioeconomic status, (3) living environment, and (4) contextual/institutional background were included in regression analysis. As demographic and family structure variables, (a) gender, (b) age and its square (to capture the possible nonlinearity of the relationship between age and health), (c) marital status (married, never married, or divorced/separated), (d) living with children, and (e) living with parents were considered. As socioeconomic status, (a) education attainment (primary school or below, junior high school, senior high school [including vocational school], college or higher [including university and the graduate school]), (b) *Hukou* (household registration; urban = 1), (c) work status (non-work, employed in public and private sectors, self-employed, and others [including working in agriculture industry], (d) whether having experienced retirement, and (e) household consumption per capita (quintile variables; as a proxy for household income) were considered. As for the living environment, binary variables of (a) house ownership and (b) having running water were constructed. As contextual/institutional background, (a) whether covered by public health insurance and (b) whether covered by private health insurance and. Finally, survey years (2011, 2013, and 2015) and regions (Eastern, Central, Western, and North-eastern; categorized by the National Bureau of Statistics of China [25]) were adjusted by including binary variables of each.

### Analytic strategy

In regression analysis, the following model was estimated:

$$ {H}_{it}=\alpha +\beta {SP}_{it-1}+\gamma {H}_{it-1}+{\boldsymbol{X}}_{it}\boldsymbol{\delta} +{u}_i+{\varepsilon}_{it}. $$

Here, *i* and *t* denote an individual and wave, respectively. *H* and *SP* indicate health outcome and SP, respectively, and ***X*** indicates a vector of covariates. *u*_*i*_ represents a set of time-invariant individual attributes, and *ε*_*it*_ is an error term. One-wave-lagged values, *H*_*it-1*_, was included in regression models for mental health, SRH, and ADL to adjust for the previous health status, while this term was not included in the regression model for each disease because the respondents who had been diagnosed with it in the previous wave were removed from the analysis. Using a one-wave-lagged variable (LV) of SP instead of its contemporaneous value is expected to mitigate biases due to reverse causality from health to SP. This model was referred to as LV1. By replacing *SP*_*it-1*_ by *SP*_*it-2*_ and also replacing *H*_*it-1*_ by *H*_*it-2*_, the longer-term effect of SP was examined. This model was referred to as LV2. In addition, biases due to individual heterogeneity were reduced by employing the random-effects (RE) model, which included time-invariant individual attributes (*u*_*i*_). This model was estimated only for LV1 and referred to as LV1 + RE. Two things should be mentioned here. First, the RE model could be applied only to LV1, because only three waves of CHARLS data were available. Second, fixed-effects (FE) models were not employed, because the Hausman test did not reject the null hypothesis that *u* is not correlated with the explanatory variables.

Regression models were further estimated separately for men and women, for three age groups (aged 45–59 years, 60–69 years, and 70 years and older), and for each SP type. The focus was on the estimated coefficient of SP (*β*). If SP has a positive impact on health—even after being adjusted for the previous health conditions, as well as covariates—*β* is expected to be positive. The software package Stata (Release 16) was used for the statistical analysis [26].

## Results

Table 1 summarizes the key features of the respondents. Table 2 compares the prevalence of six types of SP at baseline. The proportion was largest for ‘Interacting with friends’, followed by ‘Playing Mah-jong, chess or cards, or going to community club’, while it was smallest for ‘doing voluntary or charity work’, 51.3% of individuals engaged in at least one SP.

**Table 1:** Key features of the respondents at baseline

	All	Men	Women
	(*N* = 9645)	(*N* = 4561)	(*N* = 5084)
Proportion (%)			
Marital status			
Married	88.7	91.3	86.3
Never married	0.7	1.5	0.0
Divorced/Separated	10.6	7.2	13.6
Family arrangement			
Living with children	28.2	23.5	32.4
Living with parents	17.7	14.5	20.5
Socioeconomic status at baseline			
Educational attainment			
Primary school or below	63.5	53.3	72.6
Junior high school	22.6	28.7	17.2
Senior high school	11.1	13.9	8.7
College or above	2.8	4.1	1.6
Urban *hokou*	41.6	40.6	42.4
Work status			
Non-working	31.9	25.5	37.6
Employed	17.3	23.4	11.8
Self-employed	6.9	9.2	4.9
Others	54.2	56.9	51.7
Experienced retirement	28.2	23.5	32.4
Living environment			
Own house	87.2	87.7	86.8
Having running water	62.0	61.3	62.5
Health care insurance			
Public health insurance	91.7	92.5	91.0
Private health insurance	3.0	3.1	2.9
Regions			
East	30.9	31.1	30.8
Central	33.9	34.4	33.3
West	17.0	16.9	17.1
Northeast	18.2	17.5	18.8
Age (*M*, years)	58.7	59.5	58.1
Household consumption	7981	8239	7749
(*M*; per capita, annual, RMB)			

Note: ^a^ East = Beijing, Tianjin, Hebei, Shanghai, Jiangsu, Zhejiang, Fujian, Shandong, Guangdong, Hainan; Central = Shanxi, Anhui, Jiangxi, Hernan, Hubei, Hunan; West = Inner Mongolia, Guangxi, Chongqing, Sichuan, Guizhou, Yunnan, Tibet, Shaanxi, Gansu, Qinghai, Ningxia, Xinjiang; Northeast = Liaoning, Jilin, Heilongjiang

**Table 2:** Proportion (%) of social participation at baseline by type (*N* = 9645)

	Total	Men	Women
Interacting with friends	37.4	34.8	39.8
Playing Mahjong, chess or cards, or going to community club	19.8	24.3	15.7
Providing helps	7.0	8.2	6.0
going to a sport or other kind of club	6.6	6.5	6.7
Participating in a community-related organization	1.5	1.8	1.3
Doing voluntary or charity work	0.6	0.9	0.4
Caring for a sick or disabled adult	0.8	0.9	0.7
Participating in at least one activity	51.3	51.5	51.1

Table 3 compares health outcomes at follow-ups (in 2013 and 2015) between individuals who participated in social activities (SP group) at baseline (in 2011) and others (Non-SP group). Mental health, SRH, and ADL scores were all better for SP group than for Non-SP one at the 0.1% significance level (except SRH for men with *p* = 0.016). In contrast, no significant impact was observed for most diseases, and the onset of diabetes or high blood sugar was positively associated with SP. It should be noted, however, that these comparisons did not control for other factors.

**Table 3:** Health outcomes at follow-ups (in 2013 and 2015) by social participation (SP) at baseline (in 2011)

Health outcome (the higher, the better)	2013				2015			
	SP	Non-SP	Difference		SP	Non-SP	Difference	
	(*A*)	(*B*)	(*A* – *B*)	*p*-value	(*A*)	(*B*)	(*A* – *B*)	*p*-value
Mental health (MH)								
MH1 (range: 1–5)	2.0	1.9	0.1	< 0.001	2.0	1.8	0.1	< 0.001
MH2 (range: 10–70)	57.9	55.1	2.7	< 0.001	57.6	55.1	2.5	< 0.001
Self-rated health (SRH) (range: 1–5)	3.1	2.9	0.2	< 0.001	3.1	3.0	0.1	0.016
Activities of Daily Living (ADL)	42.9	42.1	0.8	< 0.001	42.8	41.8	1.0	< 0.001
BADL (range: 6–24)	23.6	23.3	0.3	< 0.001	23.5	23.1	0.4	< 0.001
IADL (range: 5–20)	19.6	19.1	0.4	< 0.001	19.5	19.0	0.5	< 0.001
Onset of disease (%)								
Hypertension or dyslipidaemia	29.8	27.4	2.4	0.079	29.2	28.0	1.2	0.397
Diabetes or high blood sugar	7.7	5.4	2.2	0.003	7.8	7.0	0.8	0.320
Heart attack or stroke	16.4	14.8	1.5	0.166	16.0	14.3	1.7	0.140
Cancer or malignant tumour	0.9	0.8	0.1	0.825	0.7	0.8	−0.1	0.684
Emotional, nervous, psychiatric problems or memory-related disease	2.0	2.0	0.0	0.907	2.1	2.3	−0.2	0.736
Stomach or digestive disease	21.6	20.2	1.4	0.257	18.5	18.6	−0.1	0.951
Other diseases	38.1	39.0	−0.9	0.541	35.8	35.3	0.5	0.742

Table 4 presents key results of regression analysis, comparing the results across LV1, LV2, and LV1 + RE models for each health outcome and disease. SP improved mental health, especially in terms of MH2, and the magnitude of its impact was larger in LV2 than LV1 and LV1 + RE, meaning that the positive impact of SP on mental health increased over time. The positive impact of SP was found for ADL in terms of both of BADL and IADL. However, it was greater in LV1 unlike in the case of mental health. Meanwhile, SRH and most diseases were insensitive to SP. No substantial difference in the results was found between LV1 and LV1+ RE models, suggesting limited biases related to individual-level heterogeneity.

**Table 4:** Estimated impacts of SP at baseline on health outcomes^a^

Model	LV1			LV2			LV1 + RE		
	Coef.		SE	Coef.		SE	Coef.		SE
Health outcome (the higher, the better)									
Mental health									
MH1 (range: 1–5)	0.016		(0.015)	0.041		(0.021)	0.014		(0.015)
MH2 (range: 10–70)	0.820	***	(0.199)	1.187	***	(0.307)	0.776	***	(0.204)
Self-rated health (SRH) (range: 1–5)	0.018		(0.035)	0.090		(0.053)	0.032		(0.036)
Activities of Daily Livings (ADL)									
BADL (range: 6–24)	0.147	***	(0.043)	0.021		(0.065)	0.158	***	(0.043)
IADL (range: 5–20)	0.159	***	(0.035)	0.019		(0.054)	0.166	***	(0.036)
Onset of disease									
Hypertension or dyslipidaemia	−0.001		(0.001)	0.001		(0.001)	−0.000		(0.001)
Diabetes or high blood sugar	0.003		(0.004)	0.001		(0.006)	0.003		(0.004)
Heart attack or Stroke	0.003		(0.005)	0.012		(0.008)	0.002		(0.005)
Cancer or malignant tumour	0.004	*	(0.001)	0.001		(0.002)	0.003	*	(0.002)
Emotional, nervous, psychiatric problems or memory-related disease	0.001		(0.002)	−0.002		(0.004)	0.000		(0.003)
Stomach or digestive disease	−0.004		(0.005)	−0.004		(0.008)	−0.004		(0.005)
Other diseases	−0.012		(0.007)	0.003		(0.010)	−0.014	*	(0.007)

Note: ^a^ Controlled for one-wave-lagged (for LV1 and LV1 + RE) or two-lagged (for LV2) wave-lagged health outcome, as well as covariates

^***^p < 0.001, ^**^p < 0.01, ^*^p < 0.05

Tables 5 and 6 provided more detailed results for health outcomes based on LV2 models, focusing on mental health, SRH, and ADL. Table 5 compared the estimation results by gender and age group for each health outcome to examine the relevance of gender- and age-related heterogeneity. As shown in Table 5, favourable impacts of SP on mental health were greater for women than men, as well as for individuals aged 60–69 compared to those in other age groups. No significant difference between genders or age groups was observed for SRH or ADL. Table 6 compares the impacts on health outcomes by SP type, focusing on MH2, BADL and IADL. As seen in this table, playing Mah-jong, chess, or cards, or going to the community club had the most substantial impact on health outcomes.

**Table 5:** Estimated impacts of SP on health outcomes by gender and age group^a^

Health outcome	Gender						Age group							
	Men			Women			Aged 45–59			Aged 60–69			Aged 70+	
	Coef.		SE	Coef.		SE	Coef.		SE	Coef.		SE	Coef.	SE
Mental health														
MH1 (range: 1–5)	0.016		(0.030)	0.060	*	(0.029)	0.070	*	(0.033)	0.011		(0.034)	0.068	(0.044)
MH2 (range: 10–70)	0.776		(0.417)	1.624	***	(0.453)	0.807		(0.476)	1.851	***	(0.499)	0.706	(0.686)
Self-rated health (RH) (range: 1–5)	0.078		(0.075)	0.090		(0.076)	0.110		(0.085)	0.013		(0.088)	0.151	(0.116)
Activities of daily livings (ADL)														
BADL (range: 6–24)	0.221	*	(0.110)	−0.113		(0.080)	0.078		(0.092)	0.028		(0.099)	− 0.059	(0.149)
IADL (range: 5–20)	0.078		(0.070)	−0.036		(0.082)	−0.009		(0.060)	0.144		(0.083)	−0.116	(0.159)

Note: ^a^ Based on LV2 models, controlled for two-wave-lagged health outcome, as well as covariates

^***^p < 0.001, ^**^p < 0.01, ^*^p < 0.05

**Table 6:** Estimated impacts of SP on health outcomes by SP type^a^

	MH2			BADL		IADL		
	(range: 10–70)			(range: 6–24)		(range: 5–20)		
	Coef.		SE	Coef.	SE	Coef.		SE
Interacting with friends	0.387		(0.315)	−0.023	(0.067)	−0.059		(0.055)
Playing Mahjong, chess or cards, or going to community club	1.167	**	(0.382)	0.133	(0.085)	0.174	**	(0.067)
Providing helps	1.263	*	(0.582)	0.033	(0.137)	0.036		(0.101)
Going to a sport or other kind of club	1.271		(0.662)	0.173	(0.153)	0.262	*	(0.115)
Participating in a community-related organization	1.294		(1.162)	0.265	(0.273)	0.116		(0.203)
Doing voluntary or charity work	−0.185		(1.749)	0.066	(0.450)	0.290		(0.306)
Caring for a sick or disabled adult	0.059		(1.826)	0.308	(0.452)	0.044		(0.320)

Note: ^a^ Based on LV2 models, controlled for two-wave-lagged health outcome, as well as covariates

^***^p < 0.001, ^**^p < 0.01, ^*^p < 0.05

## Discussion

Using the longitudinal data in China, this study examined the impact of SP on health among middle-aged adults and the elderly in China. Both the descriptive and regression analyses showed that the engagement in SP had a positive impact on health outcomes in later waves. These results were generally in line with those in previous studies that dealt with related issues in China [21–23] as well as outside the country [1–14, 27].

Among others, four findings are noteworthy. First, the positive impact of SP was remarkable for mental health as well as ADL, a result consistent with the findings in the previous studies in the developed countries [6–11]. The number of patients with mental health disorder has been increasing in China in recent years. Mental health disorder is likely to reduce the labour force participation of middle-aged adults, increase the expenditure of health care for both households and the government, and even harm the health status of family members. It is advisable for the Chinese government to encourage middle-aged and elderly adults to engage in SP to reduce the risk of mental health problems.

Second, the impact of SP on health status differed by SP type; the positive effect of SP was greatest for playing Mah-jong, chess, or cards, or going to the community club and providing helps. Playing Mah-jong, which is transitional fun in China, may increase friendships with other players and have favourable impacts on health [22]. Regarding altruistic activities, studies [28, 29] showed that such examinations enhanced the well-being of individuals, which may likely improve health outcomes. Therefore, policy measures to improve communications in a community, increase contractions with others, and encourage individuals to provide helps to others by supporting NPO activities may contribute to improving health outcomes.

Third, the positive effect of SP on health was greater for women than men. The difference may reflect the gender gap, regarding the responsibility of homework and the allocation of time between market work and homework [30, 31]. Women’s higher responsibility of homework and more time spent on it are likely to make their health more sensitive to SP outside of the home.

Lastly, the preventive impact of SP on health was greater for individuals aged 60–69 than those who were younger or older. In China, the eligible retirement age is 50 years for female workers, 55 years for female cadres, and 60 years for male workers and cadres. Thus, most individuals aged 60–69 have experienced only a few years since retirement, and their health is likely to be exposed to the isolation between the workplace or society.

This study had several limitations, as follows. First, as in most previous studies, the focus was on how baseline SP affected health outcomes in follow-up years, which led us to disregard the influence of the change of SP on health. This limitation is expected to become more serious if longitudinal data obtained from longer follow-up years are available. Second, related to the first point, the two-way causation between SP and health was not fully investigated, even if the LV method was used. Engagement in SP will improve health, which in turn will promote SP. This positive feedback seems to have amplified the observed association between baseline SP on health outcomes at follow-ups. Third, CHARLS dataset consists of individuals aged 45 years and older at baseline, leaving the association between SP and health among individuals younger than 45 years unexamined. These limitations suggest the need for more in-depth analysis using the data obtained from more waves of CHARLS in the future.

## Conclusions

The results of this study indicated a positive, albeit selective, impact of SP on health outcomes among middle-aged and elderly adults in China; SP improved mental health and ADL but did not affect SRH or diagnosed diseases, and the impact of SP differed by SP type, gender, and age. These findings provide new insights into the understanding of the association between SP and health outcomes and suggest that policy measures to encourage middle-aged and elderly adults to engage in SP are needed to enhance their health.

## Abbreviations

ADL: Activities of daily living
BADL: Basic activities of daily living
CES-D: Center for Epidemiologic Studies Depression Scale
CHARLS: Chinese Health and Retirement Longitudinal Study
FE: Fixed effects
IADL: Instrumental activities of daily living
LV: Lagged variable
SP: Social participation
SRH: Self-rated health
RE: Random effects

## References

[1] Asida T, Kondo N, Kondo K. Social participation and the onset of functional disability by socioeconomic status and activity type: the JAGES cohort study. Prev Med 2016;89:121–128. http://dx.doi.org/10.1016/j.ypmed.2016.05.006. 10.1016/j.ypmed.2016.05.006 27235600

[2] Fekete C, Brinkhof MWG, Tough H, Siegris J. Longitudinal study of social participation and well-being among persons with spinal cord injury and their partners (pro-WELL). BMJ Open 2017;7(1):e011597. http://dx.doi.org/10.1136/bmjopen-2016-011597. 10.1136/bmjopen-2016-011597 PMC5278270 28122827

[3] James BD, Wilson RS, Barnes LL, Bennett DA. Late-life social activity and cognitive decline in old age. J Inter Neur Soci 2011;17(6):998–1005. http://dx.doi.org/10.1017/S1355617711000531. 10.1017/S1355617711000531 PMC3206295 22040898

[4] Kanamori S, Kai Y, Aida J, Kondo K, Kawachi I, Hirai H (2014). Social participation and the prevention of functional disability in older Japanese: the JAGES cohort study. PLoS One.

[5] Tomioka K, Kurumatani N, Hosoi H. Association between social participation and 3-year change in instrumental activities of daily living in community-dwelling elderly adults. J Amer Geria Soci 2017;65(1):107–113. http://dx.doi.org/10.1111/jgs.14447. 10.1111/jgs.14447 27673582

[6] Anaby D, Miller WC, Eng JJ, Jarus T, Noreau L, ACC Research Group. Participation and well-being among older adults living with chronic conditions. Soc Ind Res 2011;100(1)171–183. http://dx.doi.org/10.1007/s11205-010-9611-x. 10.1007/s11205-010-9611-x PMC4478083 26120239

[7] Bourassa KJ, Memel M, Woolverton C, Sbarra DA. Social participation predicts cognitive functioning in aging adults over time: comparisons with physical health, depression, and physical activity. Aging Ment Health 2017;21(2):133–146. http://dx.doi.org/10.1080/13607863.2015.1081152. 10.1080/13607863.2015.1081152 26327492

[8] Chiao C, Weng LJ, Botticello AL. Social participation reduces depressive symptoms among older adults: an 18-year longitudinal analysis in Taiwan. BMC Public Health 2011;11:292. http://dx.doi.org/10.1186/1471-2458-11-292. 10.1186/1471-2458-11-292 PMC3103460 21569285

[9] Fu R, Noguchi H, Tachikawa H, Aiba M, Nakamine S, Kawamura A. Relation between social network and psychological distress among middle-aged adults in Japan: evidence from a national longitudinal survey. Soc Sci Med 2017;175:58–65. http://dx.doi.org/10.1016/j.socscimed.2016.12.043. 10.1016/j.socscimed.2016.12.043 28056384

[10] Gilmour H (2012). Social participation and the health and well-being of Canadian seniors. Health Rep.

[11] Pavlova MK, Silbereisen RK, Sijko K. Social participation in Poland: links to emotional wellbeing and risky alcohol consumption. Soc Ind Res 2014;117:29–44. http://dx.doi.org/10.1007/s11205-013-0332-9.

[12] Hsu HC. Does social participation by the elderly reduce mortality and cognitive impairment? Aging Ment Health 2007;11(6):699–707. http://dx.doi.org/10.1080/13607860701366335. 10.1080/13607860701366335 18074257

[13] Glass TA, de Leon CM, Marottoli RA, Berkman LF. Population-based study of social and productive activities as predictors of survival among elderly Americans. BMJ 1999;319(7208):478–483. http://dx.doi.org/10.1136/bmj.319.7208.478. 10.1136/bmj.319.7208.478 PMC28199 10454399

[14] Väänänen A, Murray M, Koskinen A, Vahtera J, Kouvonen A, Kivimäki M (2009). Engagement in cultural activities and cause-specific mortality: prospective cohort study. Prev Med.

[15] Levasseur M, Richard L, Gauvin L, Raymond E. Inventory and analysis of definitions of social participation found in the aging literature: proposed taxonomy of social activities. Soc Sci Med 2010;71(12):2141–2149. http://dx.doi.org/10.1016/j.socscimed.2010.09.041. 10.1016/j.socscimed.2010.09.041 PMC3597625 21044812

[16] Lei X, Shen Y, Smith JP, Zhou G. Do social networks improve Chinese adults’ subjective well-being? J Econ Ageing 2015;6(2015):57–67. http://dx.doi.org/10.1016/j.jeoa.2015.07.001. 10.1016/j.jeoa.2015.07.001 PMC4669891 26644993

[17] Li T, Zhang Y. Social network types and the health of older adults: exploring reciprocal associations. Soc Sci Med 2015;130(2015):59–68. http://dx.doi.org/10.1016/j.socscimed.2015.02.007. 10.1016/j.socscimed.2015.02.007 25681715

[18] Lu N, Peng C. Community-based structural social capital and depressive symptoms of older urban Chinese adults: the mediating role of cognitive social capital. Arch Geron Geria 2019;82:74–80. http://dx.doi.org/10.1016/j.archger.2019.01.014. 10.1016/j.archger.2019.01.014 30716681

[19] Norstrand JA, Xu Q. Social capital and health outcomes among older adults in China: the urban-rural dimension. Gerontologist 2012;52(3):325–334. http://dx.doi.org/10.1093/geront/gnr072. 10.1093/geront/gnr072 21746837

[20] Cao W, Li L, Zhou X, Zhou C. Social capital and depression: evidence from urban elderly in China. Aging Ment Health 2005;19(5):418–429. http://dx.doi.org/10.1080/13607863.2014.948805. 10.1080/13607863.2014.948805 25155221

[21] Liu J, Rozelle S, Xu Q, Yu N, Zhou T. Social engagement and elderly health in China: evidence from the China health and retirement longitudinal survey (CHARLS), Int J Environ Res Public Health 2019;16(2):278. http://dx.doi.org/10.3390/ijerph16020278. 10.3390/ijerph16020278 PMC6352065 30669415

[22] Gao M, Sa Z, Li Y, Zhang W, Tian D, Zhang S, Gu L. Does social participation reduce the risk of functional disability among older adults in China? A survival analysis using the 2005-2011 waves of the CLHLS data. BMC Geriatr 2018;18(1):224. http://dx.doi.org/10.1186/s12877-018-0903-3. 10.1186/s12877-018-0903-3 PMC6151053 30241507

[23] Tu R, Inoue Y, Yazawa A, Hao X, Cai G, Li Y, Lin X, He F, Yamamoto T. Social participation and the onset of hypertension among the middle-aged and older population: evidence from the China health and retirement longitudinal study. Geriatr Gerontol Int 2018;18(7):1093–1099. http://dx.doi.org/10.1111/ggi.13317. 10.1111/ggi.13317 29602268

[24] Boey KW (1999). Cross-validation of a short form of the CES-D in Chinese elderly. Intl J Geriatr Psychiatry.

[25] National Bureau of Statistics of China. Definitions of East, Central, West, and Northeast areas. http://www.stats.gov.cn/ztjc/zthd/sjtjr/dejtjkfr/tjkp/201106/t20110613_71947.htm (2020). Accessed 2 Feb 2020.

[26] StataCor. Stata Statistical Software: Release 16. College Station, TX: StataCorp LLC; 2019.

[27] Amagasa S, Fukushima N, Kikuchi H, Oka K, Takamiya T, Odagiri Y, Inoue S. Types of social participation and psychological distress in Japanese older adults: a five-year cohort study. PLoS One. 2017;12(4):e0175392. http://dx.doi.org/10.1371/journal.pone.0175392. 10.1371/journal.pone.0175392 PMC5384679 28388661

[28] Sulemana I (2015). An empirical investigation of the relationship between social capital and subjective well-being in Ghana. J Hap Stud.

[29] Woodyard A, Grable J (2014). Doing good and feeling well: exploring the relationship between charitable activity and perceived personal wellness. Voluntas.

[30] Gronau R (1997). Leisure, home production, and work: the theory of the allocation of time revisited. J Pol Econ.

[31] Ma X, Piao X (2019). The impact of intra-household bargaining power on happiness of married women: evidence from Japan. J Hap Stud.

